# Long-Term Oral Administration of Hop Flower Extracts Mitigates Alzheimer Phenotypes in Mice

**DOI:** 10.1371/journal.pone.0087185

**Published:** 2014-01-29

**Authors:** Norio Sasaoka, Megumi Sakamoto, Shoko Kanemori, Michiru Kan, Chihiro Tsukano, Yoshiji Takemoto, Akira Kakizuka

**Affiliations:** 1 Laboratory of Functional Biology, Kyoto University Graduate School of Biostudies, Sakyo-ku, Kyoto, Japan; 2 Department of Organic Chemistry, Kyoto University Graduate School of Pharmaceutical Sciences, Sakyo-ku, Kyoto, Japan; Graduate School of Pharmaceutical Sciences, The University of Tokyo, Japan

## Abstract

Coincident with the expanding population of aged people, the incidence of Alzheimer disease (AD) is rapidly increasing in most advanced countries. At present, no effective prophylactics are available. Among several pathological mechanisms proposed for AD, the “amyloid hypothesis” has been most widely accepted, in which accumulation or deposition of Aβ is considered to be the initial event. Thus, prevention of Aβ production would be an ideal strategy for the treatment or prevention of AD. Aβ is produced via the proteolytic cleavage of its precursor protein, APP (amyloid precursor protein), by two different enzymes, β and γ-secretases. Indeed, inhibitors against either or both enzymes have been developed and tested for clinical efficacy. Based on the “amyloid hypothesis”, we developed a luciferase-based screening method to monitor γ-secretase activity, screened more than 1,600 plant extracts, most of which have long been used in Chinese medicine, and observed that Hop extracts significantly inhibit Aβ production in cultured cells. A major component of the inhibitory activity was purified, and its chemical identity was determined by NMR to be Garcinielliptone HC. *In vivo*, oral administration of Hop extracts to AD model mice decreased Aβ depositions in the cerebral cortex of the parietal lobe, hippocampus, and artery walls (amyloid angiopathy) in the brains. In a Morris water maze test, AD model mice that had daily consumed Hop extracts in their drinking water showed significant mitigation of memory impairment at ages of 9 and 12 months. Moreover, in the open field test oral administration of Hop extracts also prevented an emotional disturbance that appeared in the AD mice at 18 months. Despite lifelong consumption of Hop extracts, no deleterious side effects were observed at any age. These results support the “amyloid hypothesis”, and indicate that Hop extract is a promising candidate for an effective prophylactic for AD.

## Introduction

Alzheimer Disease (AD) is the most frequently observed neurodegenerative disorder, in Japan and other advanced countries [1], [2], [3]. Several inhibitors for cholinesterase, e.g. donepezil (Aricept), are available for mitigating clinical phenotypes, but are not able to inhibit the progression of neuronal cell death; thus, the long-term prognosis has not appreciably changed [4], [5]. In the brains of AD patients, two major pathologies have been observed, namely Aβ depositions and neurofibrillary tangles [1]. Aβ is a small peptide, 40 or 42 amino acids in length, which is derived from APP (amyloid precursor protein) [6] through cleavage by β- and γ-secretases [7], [8]. Neurofibrillary tangles are composed of hyper-phosphorylated tau proteins [9].

Several pathological mechanisms have been proposed for AD. Among them, and most widely accepted, is the “amyloid hypothesis”, which posits that accumulation/deposition of Aβ is the initial event, which in turn induces neurofibrillary tangles, leading to neuronal dysfunction and neuronal cell death [10], [11]. The “amyloid hypothesis” has been supported by several clinical observations [12]. First, the *APP* gene itself, which is located on human chromosome 21, is responsible for one of the familial forms of AD with dominant inheritance [13], [14], [15]. Two other loci for familial forms of AD with dominant inheritance, on chromosome 14 and 1, have been shown to encode two related proteins, presenilin 1 and 2, respectively, and both are now known to be components of γ-secretase [16], [17]. All identified APP and presenilin mutants from AD patients produce more Aβ42 or aggregation-prone mutated Aβ than normal APP and presenilins, respectively [18], [19]. Furthermore, a recent cohort study in Iceland identified AD resistant pedigrees. These people possess a novel amino acid substitution (A673T) in APP, near the β-secretase cleavage site, resulting in decreased Aβ production [20].

Based on the “amyloid hypothesis”, several strategies to decrease Aβ production/accumulation have been tried, but any clinically successful therapeutic method or drug has not been reported. Even in the brains of healthy individuals, Aβ deposition starts in the forties [21]. It may take 20 years or more to complete the deposition, then another 20 years or more to manifest MCI (mild cognitive impairment), with a wide range of variability [22]. In AD patients, these processes tend to proceed rapidly, eventually leading to “dementia” as early as the fifties [23]. Thus, prophylactic drugs for reducing Aβ production, if available, would be best taken as early as the forties, and should be continued for the next several decades. Thus, for such prophylactic drugs, safety and lack of side effects is a critical requirement. From this perspective, we assumed that plant extracts used in Chinese medicine would be good candidates, because they have been taken by humans for more than a thousand years and are basically safe for humans when administered in moderate doses. In this study, we found that Hop flower extracts partially inhibit Aβ production, and that continuous oral administration of Hop flower extracts ameliorates not only Aβ deposition but also memory and emotional impairments of AD model mice, with no obvious side effects.

## Materials and Methods

### Cell culture and transfection

HEK293A cells were grown at 37°C in Dulbecco's modified Eagle's medium supplemented with 10% fetal bovine serum. Plasmid transfection was carried out using Lipofectamine plus (Invitrogen), according to the manufacturer's protocol. Cells in a 24-well dish were co-transfected with 200 ng each, of the plasmids pCMX-FLAG-βCTF (wild-type, V717F (Indiana mutation) [14], or V717I (London mutation) [15])-Gal4VP16, pCMX-β-galactosidase, and pTK-(GalRE)x4-Luc [24]. For evaluating Notch-cleaving activities, we transfected 200 ng pCMX-caNotch1-Gal4VP16, in which a constitutive active Notch1 fragment [25] was fused with Gal4VP16 at the C-terminus. All luciferase values, except for those from Notch1 cleavage, were obtained from the transfection of pCMX-FLAG-βCTF(V717F)-Gal4VP16. For western blotting, HEK293A cells were transfected with 300 ng of pCMX-FLAG-βCTF(V717F)-Gal4VP16 and 300 ng of pCMX-GFP. Twenty-four hours after transfection, DAPT, Hop extracts, or the purified compound was added, and then incubated an additional 24 hours. 10 µg of cell lysates were analyzed by western blot. Anti-FLAG, anti-GAL4, anti-GFP, and anti-actin were purchased from Sigma-Aldrich (MO, USA), Abcam (MA, USA), Nacalai Tesque (Kyoto, Japan), and Millipore (MA, USA), respectively.

### Ethanol extracts of plants

Ethanol extracts of plants used in Chinese medicine were purchased from an import company.

### Luciferase and β-galactosidase assays

Twenty-four hours after transfection, different amounts of test compounds in vehicle (DMSO or methanol) or vehicle alone were added to the culture medium, and then incubated for an additional 24 hours. Then, cells were harvested, whole cell extracts were prepared, and luciferase and β-galactosidase assays were carried out, as described previously [26]. The obtained raw luciferase activities were normalized by the β-galactosidase activities to compensate for different transfection efficiencies.

### ELISA

To quantify the amounts of Aβ40 and Aβ42, harvested culture media and whole cell extracts were prepared from the transfected HEK293A cells. The culture media was assayed using a human β amyloid (1–40) or (1–42) ELISA kit (WAKO), and the values obtained were normalized by the β-galactosidase activities of the corresponding cell extracts.

### Bligh-Dyer method

The Bligh-Dyer method was performed, as described previously [27], [28]. Briefly, 100 mg Hop extract was mixed with 3.8 ml of chloroform: methanol: water (1 ml: 2 ml: 0.8 ml). To the resulting mixture, 1 ml of chloroform was added and mixed, and then 1 ml of water was added and mixed. The mixture was centrifuged at 1,500 rpm for 10 min. The upper layer (water-soluble fraction: Fraction 1) and the lower layer (lipid-soluble fraction: Fraction 2) were separately collected. Both fractions were dried and weighed. The yields were 23 mg and 76 mg in Fraction 1 and Fraction 2, respectively.

### Solid-phase extraction

The dried lipid-soluble fraction (Fraction 2) from the Bligh-Dyer method was dissolved in hexane: chloroform (50∶50), and was applied to a Sep-Pak Vac 35 cc (10 g) silica cartridge (Waters). Separation procedures are described in **Figure legends**.

### HPLC purification

HPLC was performed using an Alliance 2690 HPLC system (Waters) with columns of COSMOSIL 5CN-MS (10×250 mm) (Nacalai Tesque), COSMOSIL 5C18-AR-II (10×250 mm) (Nacalai Tesque), Symmetry Shield C18 (4.6×250 mm) (Waters), and COSMOSIL π-NAP (4.6×250 mm) (Nacalai Tesque). Separation procedures for each column are described in the respective **Figure legends**.

### LC/MS analyses

The purified compound was analyzed by LC/MS using a 2795 separation module/Thermo Finnigan, LCQ Deca XP plus (Waters) [29], [30].

### NMR analysis

NMR analysis was performed using a JNM-LA 500 (JEOL). The values obtained were the following: ^1^H NMR (500 MHz, CDCl_3_) δ 4.81–4.76 (m, 3H), 3.64 (m, 1H), 2.93 (dd, *J* = 14.4, 10.9 Hz, 1H), 2.82 (dd, *J* = 14.4, 8.0 Hz, 1H), 2.70-2.59 (m, 4H), 1.57 (s, 6H), 1.52 (s, 6H), 1.30 (s, 3H), 1.24 (s, 3H), 1.23 (d, *J* = 6.3 Hz, 3H), 1.21 (d, *J* = 6.9 Hz, 3H); ^13^C NMR (125 MHz, CDCl_3_) δ 204.8, 202.3, 199.3, 158.1, 135.2, 135.0, 118.1, 117.7, 108.9, 102.3, 92.5, 72.0, 61.8, 39.2, 37.4, 34.9, 26.5, 25.8, 25.7, 25.1, 24.3, 20.1, 18.8, 17.9, 17.7. The calculated value of HRMS (FAB) C_25_H_37_O_5_ [(M+H)^+^] was 417.2641, and the observed value was 417.2632.

### Transgenic mice

The AD mice were established following a standard procedure using the C57BL/6 strain [29], [31]. The mice expressed a FLAG-tagged C-terminal portion of human APP (βCTF) with the Indiana mutation (V717F) in neuronal cells, driven by a neuron-specific enolase (NSE) promoter. The homozygous transgenic mice (V717F mice) were maintained and used for experiments. We also created another AD model mouse by co-injecting two NSE promoter-driven transgenes, one for FLAG-βCTF(V717F) and the other for HA-tagged full-length mutant presenilin 1 (P267S) [32]. The heterozygous transgenic mice (V717F/P267S mice) were used for experiments, as noted in the Results and figure legends. These mice are available via CARD (Center for Animal Resources and Development) in Kumamoto University on request. All animal studies were approved by the Review Board of Kyoto University.

### Real-time RT-PCR

Total mouse brain RNA was purified from 9-month old mice (n = 3 for each genotype), using TRIsure (BIOLINE), following the manufacturer's protocol. 300 ng of the purified RNA was used for cDNA synthesis with random primers and M-MLV reverse transcriptase (Promega). Real-time quantitative PCR was performed on a LightCycler System (Roche Diagnostics) using DNA Master SYBR Green 1 (TOYOBO). The PCR primers used were the following:

mouse GAPDH/F: 5′-CCTGCACCACCAACTGCTTA-3′

mouse GAPDH/R: 5′-TGAGCCCTTCCACAATGCCAAA-3′

human APP/F: 5′-GAAGAAGAAACAGTACACATCCAT-3′

human APP/R: 5′-CCGTTCTGCTGCATCTTGGA-3′.

### Morris water maze behavioral analysis

Morris water maze behavioral analysis was performed as described previously [33]. An escape platform (6×6 cm) was submerged 0.5 cm under the surface of the 21–22°C water in a circular tank (0.7 m in diameter and 0.2 m high). A DCR-TRV20 (SONY) camera was placed above the tank, and recorded the movements of the mice. The first 2 days of testing consisted of non-spatial training and acclimating the mice to the water and the platform. The next 5 days consisted of memory training with 4 trials each day, with the platform placed in the same quadrant. The latencies were determined by measuring the time to reach the platform. Twenty-four hours after the final trial, the platform was removed and the mice were given probe trials to test their memory of the hidden platform. The lengths of time that mice stayed in each quadrant were calculated over the 5-minute duration of the test. The open field test was performed as described previously [34]. Briefly, each mouse was placed in the center of a circular box (75 cm in diameter) and was allowed to freely explore for 5 min, under a standard fluorescent light. Three zones were set in the box (zone 3, center of the circle, with a 15 cm diameter; zone 1, the outer-most annulus, with a width of 15 cm; zone 2, the annulus between zone 1 and zone 3, with a width of 15 cm). A DCR-TRV20 (SONY) camera was placed above the field, and recorded the movements of the mice. During the test, the time spent in zones 2 and 3 was calculated using SMART software (version 2.0, Panlab).

### Y-maze test

The maze was made of black painted wood; each arm was 40 cm in length, 12 cm in height, 5 cm in width at the bottom. The arms were oriented at 120° with respect to each other. A mouse was placed at the center of the apparatus and allowed to move freely through the maze during an 8-min session. The alternation behavior (%) was calculated as the ratio of actual alternations to possible alternations, as described [35], [36].

### Histochemical analyses

Mouse brains were fixed with 4% paraformaldehyde overnight and then embedded in paraffin, and sagittal sections were prepared with a thickness of 3 µm. The sections were treated with a monoclonal anti-Aβ antibody (1–16) diluted 1∶200, (6E10, Covance) [37]. Immune signals were detected with a Vectostain elite ABC standard kit (Vector laboratories), following the manufacturer's protocol. The stereomorphological analyses were performed on 5 randomly selected regions (40,000 µm^2^ for each) of the each section of the parietal lobe from the mouse brain (n = 3 for each genotypes) by BZ-9000 Generation II microscopy (KEYENCE) with its analysis application programs [38]. FSB staining was performed following the manufacturer's protocol [39]. Briefly, the sagittal sections of brains were deparaffinized and immersed in 0.01% FSB in 50% ethanol for 30 min. Then, the sections were differentiated in saturated Li_2_CO_3_ and rinsed in 50% ethanol. The fluorescence images were obtained with AxioVision (Zeiss).

### Statistical analysis

The statistical significance was analyzed using repeated measures two-way ANOVA followed by a Holm-Sidak test or Tukey's *post-hoc*, or Student's *t* test.

## Results

### Screening of plant extracts that inhibit Aβ production

**Figure 1:**
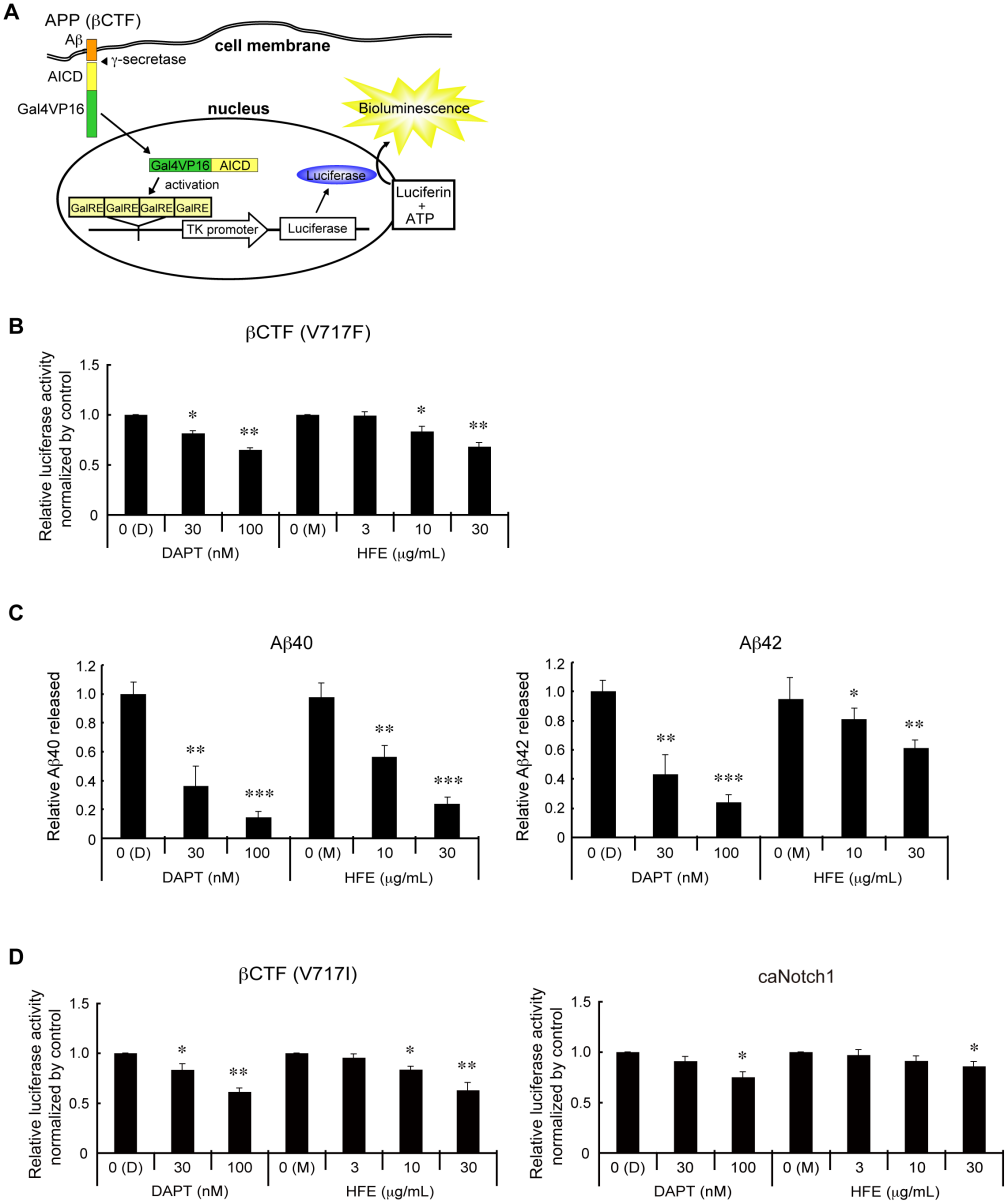
Hop extracts inhibit Aβ production. (A) Schematic drawing of the luciferase-based γ-secretase assay. The β-C-terminal fragment of APP (βCTF) was expressed in HEK293A cells as a fusion protein with Gal4VP16, a chimeric transcriptional factor, together with a luciferase-expressing reporter plasmid containing the thymidine kinase (TK) promoter with 4 copies of a Gal4-responsive element (GalRE) [24]. As an internal control of transfection, a β-galactosidase-expressing plasmid was also co-transfected. When βCTF-Gal4VP16 was cleaved by γ-secretase, the APP intracellular domain (AICD)-Gal4VP16 fusion was released and translocated into the nucleus, where it activated transcription of the luciferase reporter gene. Luciferase and β-galactosidase activities in whole cell extracts were measured. (B) Quantification of relative luciferase activities. Twenty-four hours after transfection with the plasmids for βCTF(V717F)-Gal4VP16 and β-galactosidase, HEK293A cells were treated with or without different amounts of DAPT, a commercially available γ-secretase inhibitor in DMSO (D), or HOP flower extracts (HFE) in methanol (M). Twenty-four hours after the treatments, whole cell extracts were prepared and the luciferase and β-galactosidase activities were measured. Mean values of relative luciferase activities, after normalization with β-galactosidase activities, are shown, with values for DMSO alone (D) set at 1.0. Error bars indicate standard deviations. *p<0.05, **p<0.01 by Student's *t* test. (C) Quantification of relative Aβ40 and Aβ42 amounts by ELISA. The amounts of Aβ40 and Aβ42 released into the culture medium in **B** were measured by ELISA. Mean values of relative ELISA values, after normalization with β-galactosidase activities, are shown, with values for DMSO alone (D) set at 1.0. Error bars indicate standard deviations. *p<0.05, **p<0.01, ***p<0.005 by Student's *t* test. (D) Quantification of relative luciferase activities. Twenty-four hours after transfection with plasmids expressing β-galactosidase and βCTF(V717I)-Gal4VP16 (left panel) or caNotch1-Gal4VP16 (right panel), HEK293A cells were treated with or without different amounts of DAPT in DMSO (D) or HOP flower extracts (HFE) in methanol (M). Twenty-four hours after the treatments, whole cell extracts were prepared and the luciferase and β-galactosidase activities were measured. Mean values of relative luciferase activities, after normalization with β-galactosidase activities, are shown, with values for DMSO alone (D) set at 1.0. Error bars indicate standard deviations. *p<0.05, **p<0.01 by Student's *t* test.

We previously developed an assay in which proteolytic activities directed at membrane proteins can be converted to a transcriptional activity readout [24]. We constructed a similar system to monitor the cleavage of APP by γ-secretase (**Fig. 1A**). In this system, Gal4VP16, a chimeric transcription factor, was fused to the FLAG-tagged C-terminal end of the β-C-terminal fragment of APP (βCTF), and the resulting FLAG-βCTF-Gal4VP16 fusion protein was expressed in HEK293A cells, together with a luciferase reporter plasmid, which contains 4 copies of GalRE [24], a Gal4-responsive element in the promoter region. We also co-transfected a plasmid expressing β-galactosidase for the normalization of transfection efficiencies. Twenty-four hours after the transfection, measurable levels of both luciferase and β-galactosidase were observed. In the presence of DAPT, a commercially available γ-secretase inhibitor, luciferase but not β-galactosidase activity was decreased, indicating that this system can monitor the inhibition of γ-secretase (**Fig. 1B**). By ELISA, we confirmed the decrease of Aβ production in the presence of DAPT (**Fig. 1C**).

Using this luciferase assay system, we screened ethanol-extracts of more than 1,600 plants, most of which are currently used in Chinese medicine, and found that among them Hop flower extracts had the most potent inhibitory activity toward the luciferase reporter. Hop flower extracts showed similar inhibitory activities toward the cleavage of substrates with Indiana (V717F) or London (V717I) mutations (**Fig. 1B, D**), but less efficient inhibition of Notch1 cleavage (**Fig. 1D**). The inhibitory activity was dose-dependent (**Fig. 1B, C, D**). We observed very little inhibitory activity in water-extracts of Hop flowers. These results indicated that the responsible molecule(s) is lipid-soluble. Using ELISA, we confirmed that ethanol-extracts of Hop decreased production of both Aβ40 and Aβ42 (**Fig. 1C**). These results indicated that ethanol-extracts of Hop flowers (referred to as “Hop extracts” hereafter) contain molecule(s) that inhibit γ-secretase.

### Isolation and identification of a major γ-secretase-inhibitory component from Hop extracts

**Figure 2:**
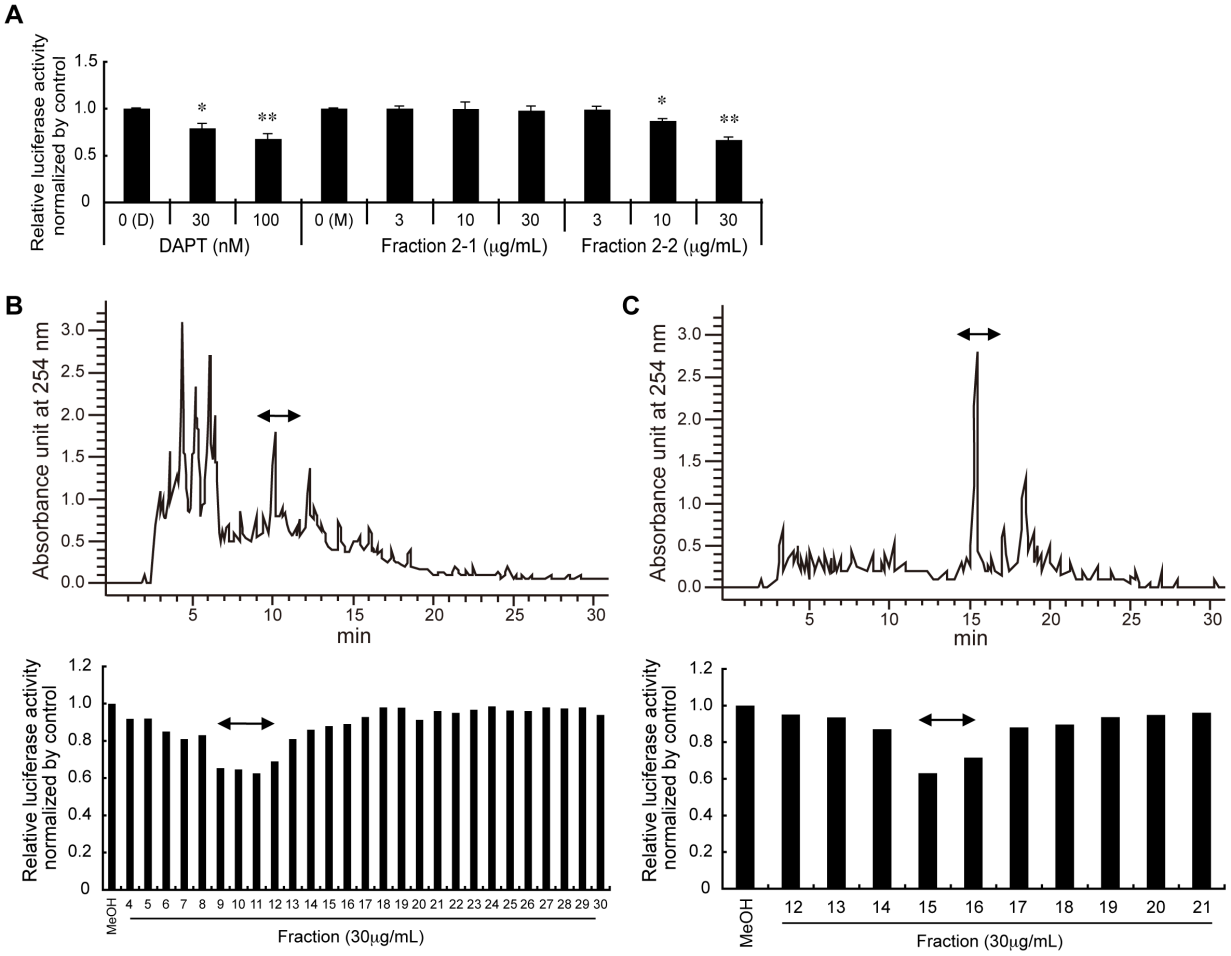
Purification of a major component in Hop extract that inhibits Aβ production. (A) Results of the luciferase assay after solid phase extraction. 330 mg of the lipid-soluble fraction (Fraction 2) from the Bligh-Dyer method was applied to a Sep-Pak Vac Silica Cartridge, and was eluted with 100% chloroform (fraction 2-1), followed by 100% methanol (fraction 2-2). Each fraction was dried and dissolved in methanol (M), then assayed for inhibition of Aβ production using the luciferase assay, as described in Fig. 1B. Mean values of relative luciferase activities, after normalization with β-galactosidase activities, are shown, with values for DMSO alone (D) set at 1.0. Bars denote standard deviations. *p<0.05, **p<0.01 by Student's *t* test. (B) A representative chromatogram from the first reverse-phase HPLC is shown (upper panel). 180 mg of Fractions 4–7 from the normal-phase HPLC (Fig. S1 and S3) were applied to a COSMOSIL 5C18-AR-II column, and were eluted with a linear gradient of acetonitrile, starting from 80% to 100% for 20 min, followed by continuous flow with 100% acetonitrile. Effluent fractions were collected every minute. The results of the luciferase assay for each fraction are shown (lower panel). Fractions 9–12 were pooled and used for further purification. (C) A representative chromatogram from the second reverse-phase HPLC is shown (upper panel). 26 mg of pooled fractions 9–12 in (B) was applied to a symmetry shield C18 column, and was eluted following the same procedures as in (B). Effluent fractions were collected at 1-minute intervals. The results of the luciferase assay for each fraction are shown (lower panel). Fractions 15–16 were pooled and used for further purification.

In order to isolate major components of Hop extracts that inhibit γ-secretase, we first performed a Bligh-Dyer separation protocol (**Fig. S1**). As expected, the inhibitory activities were detected in the lipid-soluble fraction (Fraction 2) (**Fig. S2**). Next, we separated Fraction 2 by solid-phase extraction with a Sep-Pak Vac Silica cartridge: firstly eluted by with 100% chloroform (Fraction 2-1), followed by 100% methanol (Fraction 2-2). We found that the inhibitory activities were eluted in Fraction 2-2, but not Fraction 2-1 (**Fig. 2A**). We then applied Fraction 2-2 to normal-phase HPLC analysis with a 5CN-MS column, and observed that the inhibitory activities were eluted in Fractions 4-7 (**Fig. S3**). We next applied Fractions 4–7 to reverse-phase HPLC analysis with a COSMOSIL 5C18-AR-II column, and observed that the inhibitory activities were eluted in Fractions 9–12 (**Fig. 2B**). We then applied Fractions 9–12 to additional reverse phase HPLC analysis with a Symmetry Shield C18 column, and found that the inhibitory activities were eluted in Fractions 15–16 (**Fig. 2C**).

**Figure 3:**
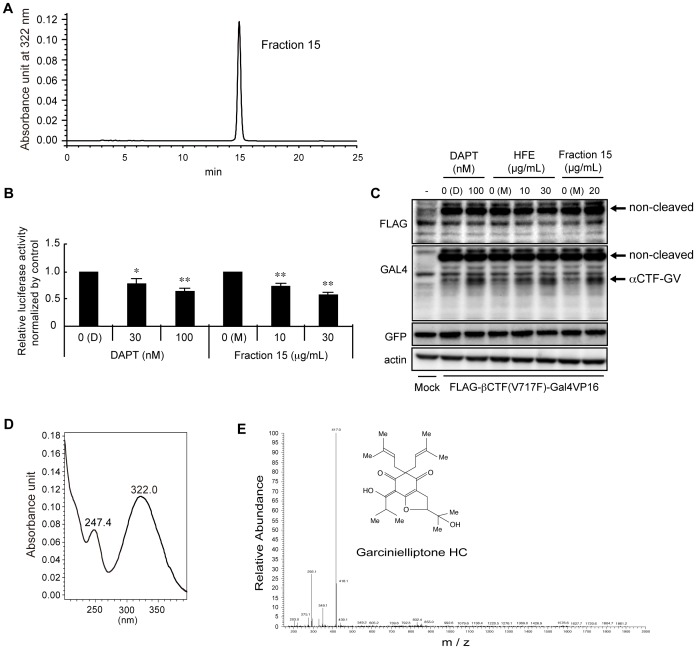
Characterizations of the purified compound. (A) A representative chromatogram from the third reverse-phase HPLC is shown (upper panel). 7.3 mg of pooled fractions 15–16 in Fig. 2C were applied to a COSMOSIL π-NAP column, and were eluted by continuous flow with 65% acetonitrile. The compound was eluted at 15 min as a single peak. (B) Comparison of DAPT and the purified compound by the luciferase assay. The estimated IC_50_ value of the purified compound was 5.4 µg/mL. (C) Comparison of DAPT, Hop extracts (HFE), and the purified compound (Fraction 15) by western blot analysis of the cleavage products of exogenously expressed FLAG-βCTF(V717F)-Gal4VP16 in HEK293A lysates. Positions of FLAG-βCTF(V717F)-Gal4VP16 (non-cleaved) and αCTF-Gal4VP16 (αCTF-GV) are shown. GFP served as an internal control of the transfection, and actin served as a loading control. (D) Optical absorbance profile of the purified compound. (E) LC/MS profile of the purified compound. The molecular mass of the purified compound was determined as 416 g/mol; obtained value of M+H was 417.2632. The molecular architecture of the purified compound, determined by NMR, is also shown. Note that the chemical formula of the compound was C_25_H_36_O_5_.

Lastly, we applied Fractions 15–16 to a COSMOSIL π-NAP reverse-phase HPLC column, and observed a single peak that eluted in Fraction 15 (**Fig. 3A**). We confirmed that the purified compound indeed retained the ability to inhibit Aβ production, as evidenced by the luciferase assay (**Fig. 3B**). The estimated IC_50_ values of the purified compound and DAPT were 5.4 µg/mL and 29 nM, respectively, the latter of which was comparable to the known IC_50_ value of DAPT (20 nM) [40]. It is notable, however, that the specific activity relative to the initial Hop extracts did not appreciably increase over the course of the purification (see discussion). By western blot, we examined the cleavage products in lysates from HEK293A cells transfected with the FLAG-βCTF(V717F)-Gal4VP16 construct. As reported previously [41], [42], it was very difficult to detect AICD. However, DAPT increased the amounts of αCTF due to the decrease of γ-cleavage. Very similar to DAPT, both Hop extracts and the purified compound increased the amounts of αCTF (**Fig. 3C**). These results further support the idea that Hop extracts and the purified compound suppress γ-secretase activity.

We also measured the optical absorbance profile of the purified compound (**Fig. 3D**). Two absorbance peaks at 247 and 332 nm were assigned to α, β- and α, β, γ, δ-unsaturated carbonyl bonds, respectively (**Fig. 3E**). We then determined its molecular mass by LS/MS to be 416 g/mole (**Fig. 3E**). Repeated purifications yielded a total of approximately 2 mg of the purified compound. We then analyzed its chemical structure by NMR and found that its NMR values perfectly fit with those of a previously reported compound, Garcinielliptone HC [43], and its chemical formula was determined to be C_25_H_36_O_5_ (**Fig. 3E**).

### Amelioration of AD phenotypes in AD model mice by continuous oral administration of Hop extracts

**Figure 4:**
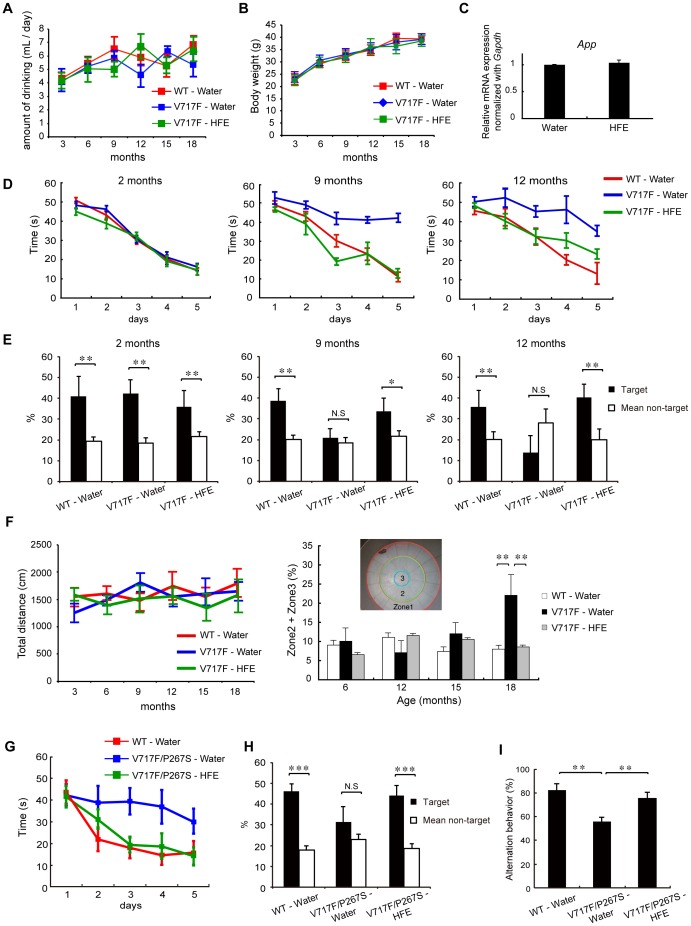
Mitigation of memory impairment in AD model mice by oral administration of Hop extracts. (A) Average daily water consumption per mouse, per experimental group. Decrease of water volume in each water bottle was measured every 7 days. No significant difference was observed in the amount of water consumed, comparing the three groups. (B) Body weights of wild-type mice, water-drinking V717F mice, and Hop extract-drinking V717F mice, from age 3 months to 18 months. Bars represent standard deviations. No significant difference was observed among the groups of mice. (C) Quantitative RT-PCR analysis of transgene mRNA in the brains of water-drinking V717F mice and Hop extract-drinking V717F mice. Brain mRNAs from 9-month old water-drinking V717F mice and Hop extract-drinking V717F mice were analyzed by quantitative RT-PCR to determine the expression levels of the transgene mRNA. No significant difference was observed among the groups. (D) Morris water maze test with V717F mice. The test measured the time required for mice to locate a hidden platform. At ages of 2 months, no significant difference was observed among the groups of mice. At ages of 9 and 12 months, Hop extract-drinking V717F mice (n = 7), required significantly shorter times to reach the hidden platform, compared with water-drinking V717F mice (n = 7). F = 11.4, p<0.001 in 9-month-old mice, F = 20.94, p<0.01 in 12-month-old mice, by two-way repeated measures ANOVA followed by a Holm-Sidak test. Bars indicate standard deviation. (E) Probe test in the Morris water maze test. At ages of 2 months, all 3 groups of mice spent significantly more time in the quadrant where the hidden platform had been placed. At ages of 9 and 12 months, water-drinking wild-type mice (n = 7) and Hop extract-drinking V717F mice (n = 7) showed a significantly prolonged stay in the quadrant where the hidden platform had been placed, but this ability was lost in water-drinking AD mice (n = 7). Bars indicate standard deviation. *p<0.05, **p<0.01 by Student's *t* test. (F) Open field test for basal activity and anxiety. At ages of 6, 12, 15, and 18 months, no significant differences in basal activity were observed among the three groups. However, at 18 months, water-drinking AD mice (n = 7) stayed a significantly longer period in zone 2 or zone 3, as compared with age-matched wild-type (n = 8) or Hop extract-drinking AD mice (n = 7). F = 6.58, **p<0.01 by repeated measures two-way ANOVA, Tukey's *post-hoc*. (G) Morris water maze test with V717F/P267S mice. At ages of 9 months, Hop extract-drinking V717F/P267S mice (n = 7), required significantly shorter times to reach the hidden platform, compared with water-drinking V717F/P267S mice (n = 5). F = 8.51, p<0.05 by two-way repeated-measures ANOVA followed by Holm-Sidak test. Bars indicate standard deviation. (H) Probe test in the Morris water maze test. At ages of 9 months, water-drinking wild-type mice (n = 8) and Hop extract-drinking V717F/P267S mice (n = 7) showed a significantly prolonged stay in the quadrant where the hidden platform had been placed, but such prolonged stay was lost in water-drinking V717F/P267S mice (n = 7). Bars indicate standard deviation. *p<0.05, **p<0.01 by Student's *t* test. (I) Y-maze test with V717F/P267S mice. At ages of 9 months, water-drinking V717F/P267S mice showed significantly higher percent alternation, compared with water-drinking wild-type or Hop extract-drinking V717F/P267S mice. Bars indicate standard deviation. **p<0.01 by Student's *t* test.

We next examined the *in vivo* effects of Hop extracts on AD model mice, in which the FLAG-tagged β-C-terminal fragment of APP (βCTF) with Indiana mutation (V717F) was expressed under the control of the NSE (neuron-specific enolase) promoter. These transgenic mice were mated to obtain homozygous mice, and then the homozygous mice were bred to achieve sufficient numbers (referred to as V717F mice hereafter). For the experiments, V717F mice were separated into two groups. In one group, mice were allowed to drink water freely. In the other group, mice were allowed to freely drink Hop extract-containing water (2 g extract per liter of water) after weaning. In both groups and age-matched wild-type mice, total amounts of water consumption did not differ, approximately 4∼6 ml/day/mouse (**Fig. 4A**). Body weights also did not differ between all 3 groups throughout the examination periods, up to 18 months of age (**Fig. 4B**). In both groups of V717F mice, expression of the transgene mRNAs in the brain were not statistically different (**Fig. 4C**), indicating that the Hop extract did not affect the NSE promoter activity or stability of the transgene mRNA.

At 2 months of age, both groups of V717F mice did not display any impairment of spatial memory in the Morris water maze test, as compared with age-matched wild-type mice (**Fig. 4D**). At 9 months of age, water-drinking V717F mice showed significant impairment in spatial memory in the Morris water maze test. These mice needed more time to reach the hidden platform, as compared with the age-matched wild-type mice. In contrast, Hop extract-drinking V717F mice were able to reach the hidden platform significantly faster than the water-drinking V717F mice. The ability of the Hop extract-drinking V717F mice to reach the platform appeared equal to or even slightly better than the age-matched wild-type mice (**Fig. 4D**). By 12 months, Hop extract-drinking V717F mice were still able to reach the hidden platform significantly faster than the water-drinking V717F mice, but apparently slower than the age-matched wild-type mice (**Fig. 4D**). In the follow-up probe test, water-drinking V717F mice spent a similar time around the area where the hidden platform had been placed, as compared to the time spent in the other area. However, the age-matched wild-type mice or Hop extract-drinking V717F mice spent significantly more time around the area where the hidden platform had been placed than in the other area, at ages of 9 as well as 12 months (**Fig. 4E**).

We noted that no significant difference in basal activities were observed in the 3 groups of mice, up to ages of 18 months (**Fig. 4F, left panel**), and thus the observed differences in time to reach the hidden platform were not due to differences in physical activities. At ages of 15 and 18 months, however, a significant difference in time to reach the hidden platform was no longer observed between water-drinking V717F mice and Hop extract-drinking V717F mice (data not shown). These results indicate that Hop extracts have the ability to delay the V717F phenotypes but not to completely prevent their manifestation. In the open field test, by which not only basal activities but also emotional states were able to be examined, all 3 groups of mice behaved indistinguishably at ages of 6, 12, and 15 months (**Fig. 4F, right panel**). At ages of 18 months, only water-drinking V717F mice displayed significantly longer times away from the walls, indicating that the water-drinking V717F mice were impaired in feeling anxiety, a type of emotional disturbance, and that Hop extracts might prevent emotional disturbances in AD as well.

We also created another transgenic AD model mouse, in which the FLAG-βCTF(V717F) and full-length mutant presenilin 1 (P267S) were co-expressed under the control of the NSE promoter. These transgenic mice were maintained as heterozygous mice (referred as to V717F/P267S mice hereafter), and were subjected to memory tests. Up to 7 months of age, V717F/P267S mice did not display any impairment of spatial memory in the Morris water maze test, as compared with age-matched wild-type mice (**Fig. S4**). At 9 months of age, however, water-drinking V717F/P267S mice showed significant impairment in spatial memory in the Morris water maze test, similar to the impairment observed in water-drinking V717F mice. These V717F/P267S mice needed more time to reach the hidden platform, as compared with the age-matched wild-type mice. In contrast, Hop extract-drinking V717F/P267S mice were able to reach the hidden platform significantly faster than the water-drinking V717F/P267S mice. The ability of the Hop extract-drinking V717F/P267S mice to reach the platform was comparable to the age-matched wild-type mice (**Fig. 4G**).

In the probe test, water-drinking V717F/P267S mice spent a similar time around the area where the hidden platform had been placed, as compared to the time spent in the other area. However, the age-matched wild-type mice or Hop extract-drinking V717F/P267S mice spent significantly more time around the area where the hidden platform had been placed than in the other area (**Fig. 4H**). Consistently, water-drinking V717F/P267S mice showed significantly reduced proficiency of special memory in a Y-maze test, as compared to the age-matched wild-type mice or Hop extract-drinking V717F/P267S mice (**Fig. 4I**).

**Figure 5:**
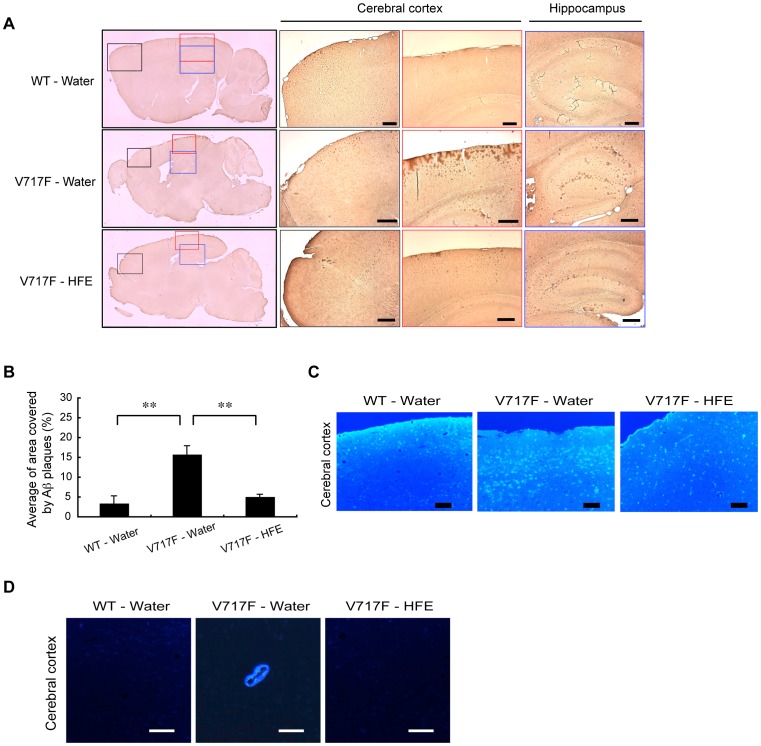
Mitigation of Aβ depositions in V717F mice by oral administration of Hop extracts. (A) Histochemical analyses of mouse brains stained by an anti-Aβ antibody. Sagittal sections of brains of 16-month old mice (n = 3 in each) were stained with an anti-Aβ antibody. Aβ depositions were visualized (brown color) by the ABC method. Scale bars, 200 µm. (B) Quantification of Aβ depositions in sections of parietal lobes (areas indicated by red boxes in **A**) with a BZ-9000 Generation II microscope (KEYENCE) and image analysis application programs. Bars indicate standard deviation. **p<0.01 by Student's *t* test. (n = 15 for each, see details in **Materials and methods**). (C) Histochemical analysis of sections in the cerebral cortex of the parietal lobe of mouse brains stained by FSB, an amyloid sensitive fluorescent dye. Scale bars, 200 µm. (D) A representative photo of amyloid angiopathy. At 16 months, the Aβ deposition in the artery wall was specifically observed in the brains of water-drinking AD mice, but not in those of age-matched wild-type and Hop extract-drinking AD mice. Scale bars, 50 µm.

Finally, we examined whether Hop extracts were indeed able to reduce Aβ depositions in the brain of our V717F model mice. We sacrificed 16-month old mice and examined their brains by immunohisotchemical analyses. On first inspection, we had the impression that the brains of water-drinking V717F mice were smaller than those of the others, but the differences were not significant. In the cerebral cortex of the frontal lobe (areas indicated by black boxes in **Fig. 5A**), we could not observe any clear difference of Aβ deposition among water-drinking V717F mice, Hop extract-drinking V717F mice, and age-matched wild-type mice. In the cerebral cortex of the parietal lobe (areas indicated by red boxes in **Fig. 5A**), very large amounts of Aβ deposition were observed in water-drinking V717F mice. However, the Aβ deposition was greatly reduced in age-matched Hop extract-drinking V717F mice. Slight Aβ deposition was observed in the cerebral cortex of the parietal lobe in the age-matched wild-type mice (**Fig. 5B and C**). Similar results were observed by FSB staining (**Fig. 5C** **and Fig. S5**). Furthermore, in the hippocampus (areas indicated by blue boxes in **Fig. 5A**), Aβ deposition was much reduced in Hop extract-drinking V717F mice compared with water-drinking V717F mice. It is notable that in water-drinking V717F mice, Aβ deposition was clearly observed around certain artery walls, albeit infrequently, which is termed “amyloid angiopathy” [44], but such “amyloid angiopathy” was barely observed in age-matched Hop extract-drinking V717F mice or wild-type mice (**Fig. 5D**). In Hop extract-drinking V717F and wild-type mice, we did not observe any obvious skin problems, e.g. acne inversa **[45]**, **[46]**, skin cancers [47], [48], etc. up to ages of 18 months (see Discussion).

## Discussion

The “amyloid hypothesis” predicts that reduction of Aβ production or accumulation would be an effective strategy for the treatment or prevention of AD. Accordingly, one approach has focused on the development of inhibitors of γ-secretase, whose activity is crucial to overall Aβ production as well as the Aβ40/Aβ42 ratio. Indeed, Semagacestat, an investigational compound, was developed and was tested in clinical trials on AD patients, but the trials did not show any significant slowing of AD phenotypes, or even worsened the symptoms in a phase III clinical study [48]. In addition, an increased risk of skin cancers has been reported [48], which might be due to inhibition of Notch signaling pathways by the γ-secretase inhibitor [47].

Recently, it has been proposed that enhanced activation of γ-secretase may promote the degradation of Aβ and thus lead to reduced Aβ accumulation [49]. An alternative approach was to neutralize Aβ by Aβ immunization or with Aβ-specific antibodies. Such biological drugs, e.g. Bapineuzumab, were developed, and their efficacies were tested in large clinical trials, but no benefit was observed [50], [51]. In addition, during anti-Aβ immunotherapies, several patients incurred intracranial hemorrhaging or meningoencephalitis as a complication, which prompted the discontinuation of the clinical trials [52]. The hemorrhaging was assumed to be caused by the Aβ antibody reacting with amyloid angiopathy.

The failures of these trials for AD treatment notwithstanding, a recent cohort study in Iceland brought new strong evidence supporting the idea that reducing Aβ indeed reduces AD risk. The study identified an AD-resistant mutation (A673T) in APP near the β cleavage site [20]. In cell culture experiments, the mutated APP produced approximately half the amount of Aβ than non-mutated APP. Thus, individuals possessing one allele of the mutated APP gene should have a 25% reduction of Aβ production [20]. Given that such individuals showed very low risk of AD, mild inhibition of Aβ production might be optimal for avoiding AD. As shown in this study, Hop-extracts contain mild activities that partially inhibit Aβ production in cultured cells, and indeed they proved to be markedly effective in preventing not only learning and memory impairment but also Aβ depositions in AD model mice. These results support the “amyloid hypothesis” and are consistent with the idea that mild inhibition of Aβ production is a plausible strategy to reduce AD risk [53]. Moreover, we did not observe any skin problems throughout the administration of Hop extracts up to 18 months, indicating that mild inhibition of γ-secretase is not deleterious.

In humans, it is generally believed that memory disturbance in AD patients occurs long after the completion of Aβ deposition. By contrast, in our V717F mice, neurological phenotypes apparently precede the completion of Aβ deposition. In 16-month old V717F mice, administration of Hop extracts was still visibly apparent as reduced Aβ deposition, but at that age, the Hop extract-drinking mice had already lost memory ability to levels similar to those of water-drinking V717F mice. These apparent differences notwithstanding, our results demonstrating that Hop extracts have the ability to reduce Aβ deposition as well as to forestall memory impairment in the AD model mice consistently support the importance of Aβ production for the onset of AD.

In this study, we also succeeded in purifying a major active component in Hop extracts that inhibits γ-secretase. As often observed in the purification procedures of plant extracts, specific activities were not substantially increased during the purification. This result raises the possibility that the inhibitory activity of the purified compound might be enhanced synergistically with other components in the hop extracts. As is often observed in Chinese medicine, the single usage of the purified compound by itself might not be effective for AD. By mass analysis, its molecular weight was determined to be 416, and its chemical structure was solved by NMR analysis and was found to be Garcinielliptone HC [43]. This compound was originally isolated from Garcinia, suggesting that Garcinia extracts might also have the potential to reduce Aβ production or accumulation. This possibility remains to be tested. Future studies are necessary to reveal the mechanism of inhibition.

In addition to the observed Aβ-reducing activity, Hop-extracts have been reported to contain other interesting properties, including anti-inflammatory activities [54], estrogen-like activities [55], [56], anti-atherosclerotic activities [57], etc. It has long been argued that chronic inflammation may be a mechanism underlying AD [58], [59], and recent clinical studies indicate that estrogen therapy reduced the risk of AD, especially for young postmenopausal women [60], [61]. These additional Hop activities might also add some benefits to the prevention or retardation of AD progression. Hop extracts have long been used in Chinese medicine for sedation, calming gastric and intestinal disorders, diuretics, etc. In Europe, Hop flower has been used as an herb for the treatment of insomnia, neuralgia, and menopausal disorders [62], [63]. Furthermore, from their estrogen-like activities, Hop extracts have been tested in several clinical trials for reducing post-menopausal complications, at a maximal dose of 300 mg/day, and no obvious side effects were reported [64].

Recently, γ-secretase mutations have been reported to cause acne inversa [45], [46]. In our mouse experiments, more than one and a half years of daily administration of Hop extracts did not manifest acne inversa nor elicit any overt deleterious effects. These lines of evidence encourage us to further investigate Hop extracts as safe and promising anti-AD substances. Finally, it should be mentioned that Hop sub-species contain different levels of γ-secretase inhibitory activities, and some have no measurable activity at all. Growing locales and conditions might also affect the activities. Thus, quality control of the extracts will be critical for maximizing the prophylactic effects on AD.

## Supporting Information

Figure S1 **A schematic flow diagram of the purification procedures.**
(TIFF)

Figure S2 **Quantification of inhibition of Aβ production by fractions from the Bligh-Dyer method.** Mean values of relative luciferase activities from cells treated with DAPT and Fractions 1 and 2 of Bligh-Dyer method, after normalization with β-galactosidase activities, are shown. Values in the absence of test compounds (0) are set at 1.0. Error bars indicate standard deviations. *p<0.05, **p<0.01
(TIFF)

Figure S3 **Quantification of inhibition of Aβ production by fractions from the first normal-phase HPLC.** A representative chromatogram of the normal-phase HPLC is shown (upper panel). 250 mg of Fraction 2-2 from the solid phase extraction (**Fig. 2A**) were applied to a COSMOSIL 5CN-MS column, and were eluted by a linear gradient of methanol, from 0% to 15% over 15 min, in hexane: chloroform (1∶1), followed by continuous flow of 15% methanol in Hexane: chloroform (1∶1). Effluent fractions were collected every minute. The results of the luciferase assay on each fraction are shown (lower panel).
(TIFF)

Figure S4 **Morris water maze test with V717F/P267S mice.** The test measured the time required for mice to locate a hidden platform. At ages of 5 and 7 months, no significant difference was observed among the groups of mice.
(TIFF)

Figure S5 **Enlarged images of FSB staining.** Sections of the cerebral cortex of the parietal lobe (CTX) and the hippocampus (HC), from 16-month old mice, were stained by FSB. Scale bars, 20 µm. Stronger FSB signals were observed in the sections from water-drinking V717F mice (V717F) than those from age-matched wild-type mice (WT).
(TIFF)
