# Supplementary figures and images for: Long-Term Oral Administration of Hop Flower Extracts Mitigates Alzheimer Phenotypes in Mice
Source: PLoS One. 2014 Jan 29;9(1):e87185. doi: 10.1371/journal.pone.0087185 (PMC3906130; doi:10.1371/journal.pone.0087185)

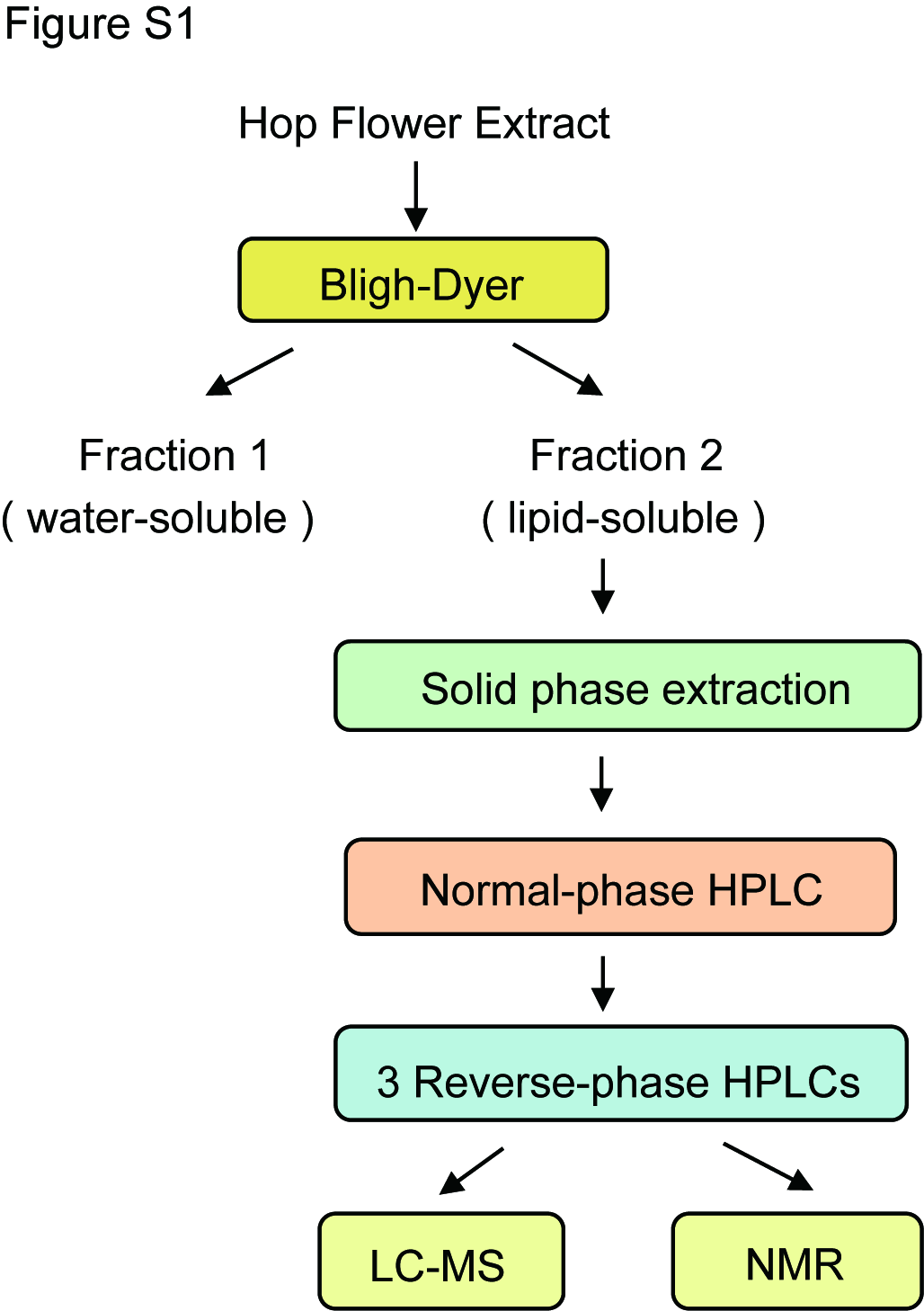

Supplement: Figure S1 — A schematic flow diagram of the purification procedures. (TIFF) [file pone.0087185.s001.tiff]

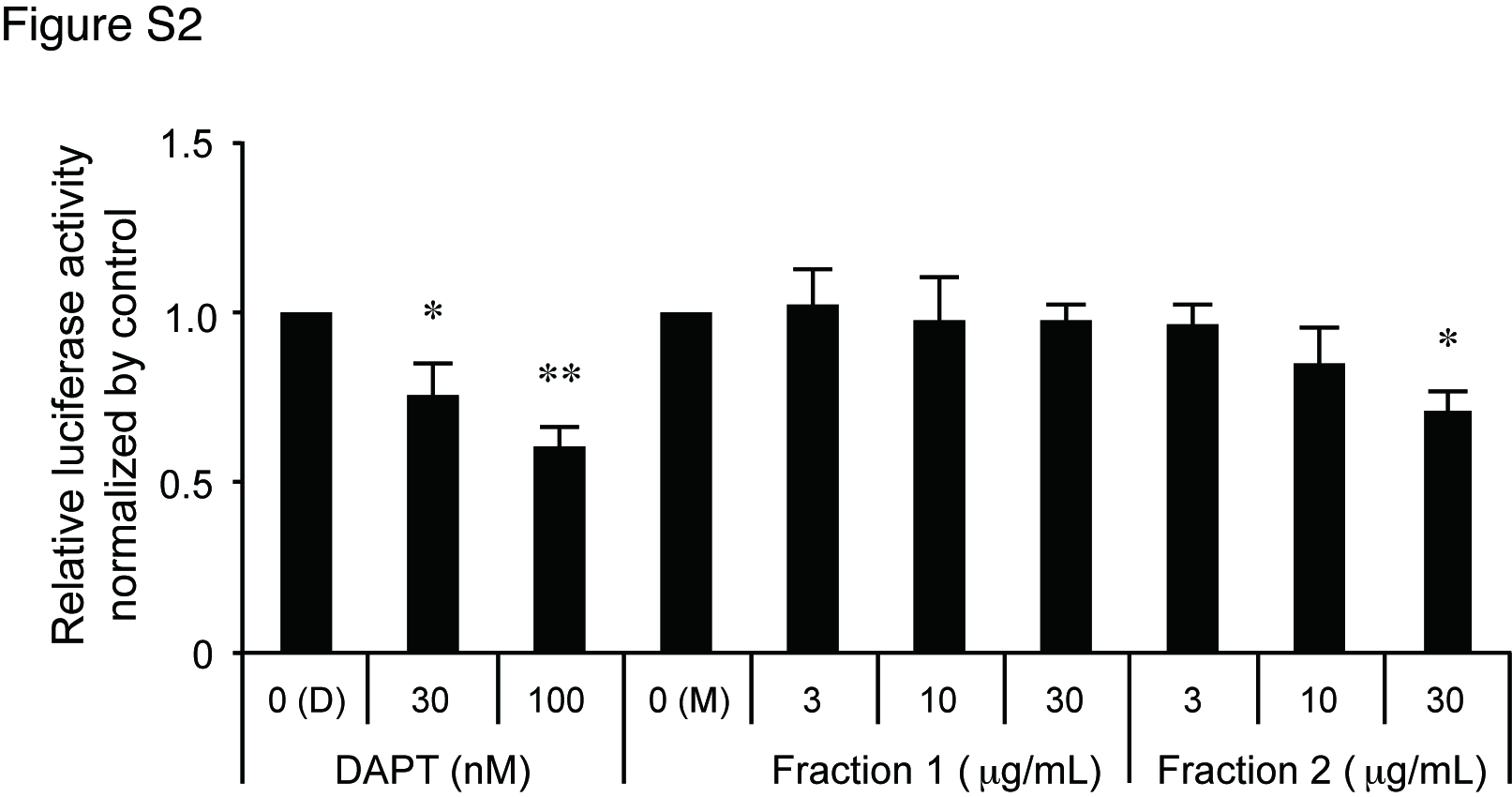

Supplement: Figure S2 — Quantification of inhibition of Aβ production by fractions from the Bligh-Dyer method. Mean values of relative luciferase activities from cells treated with DAPT and Fractions 1 and 2 of Bligh-Dyer method, after normalization with β-galactosidase activities, are shown. Values in the absence of test compounds (0) are set at 1.0. Error bars indicate standard deviations. *p<0.05, **p<0.01 (TIFF) [file pone.0087185.s002.tiff]

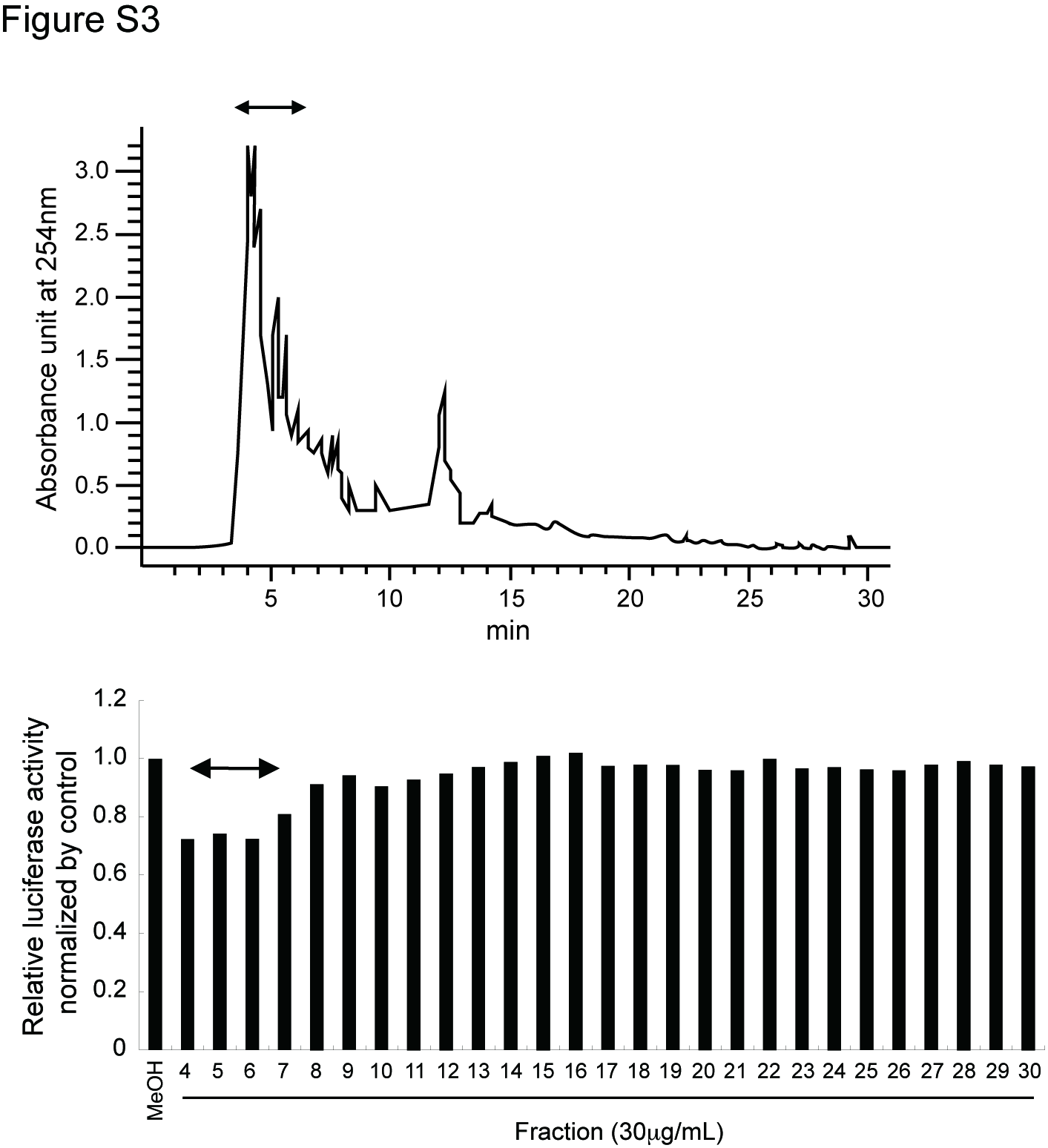

Supplement: Figure S3 — Quantification of inhibition of Aβ production by fractions from the first normal-phase HPLC. A representative chromatogram of the normal-phase HPLC is shown (upper panel). 250 mg of Fraction 2-2 from the solid phase extraction ( Fig. 2A ) were applied to a COSMOSIL 5CN-MS column, and were eluted by a linear gradient of methanol, from 0% to 15% over 15 min, in hexane: chloroform (1∶1), followed by continuous flow of 15% methanol in Hexane: chloroform (1∶1). Effluent fractions were collected every minute. The results of the luciferase assay on each fraction are shown (lower panel). (TIFF) [file pone.0087185.s003.tiff]

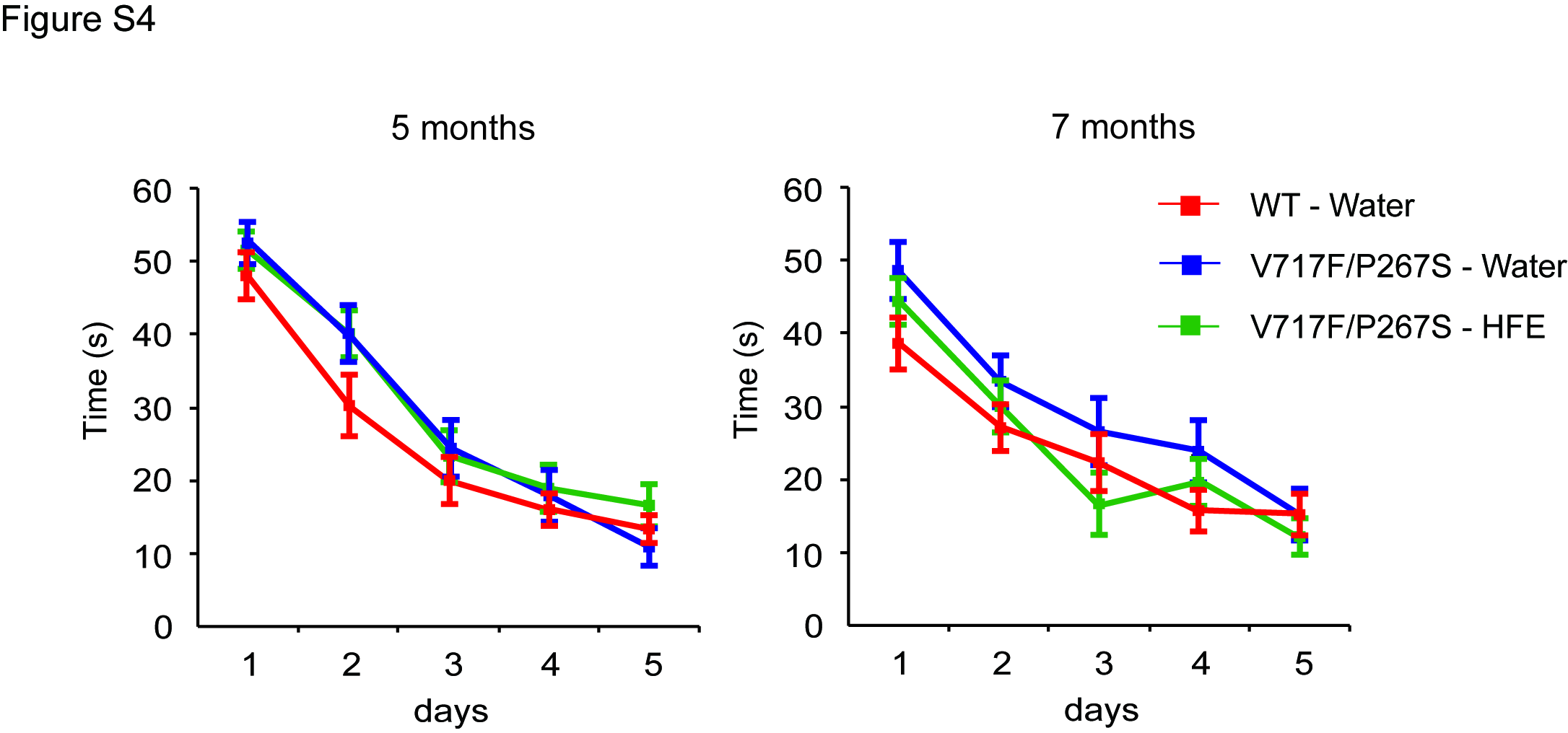

Supplement: Figure S4 — Morris water maze test with V717F/P267S mice. The test measured the time required for mice to locate a hidden platform. At ages of 5 and 7 months, no significant difference was observed among the groups of mice. (TIFF) [file pone.0087185.s004.tiff]

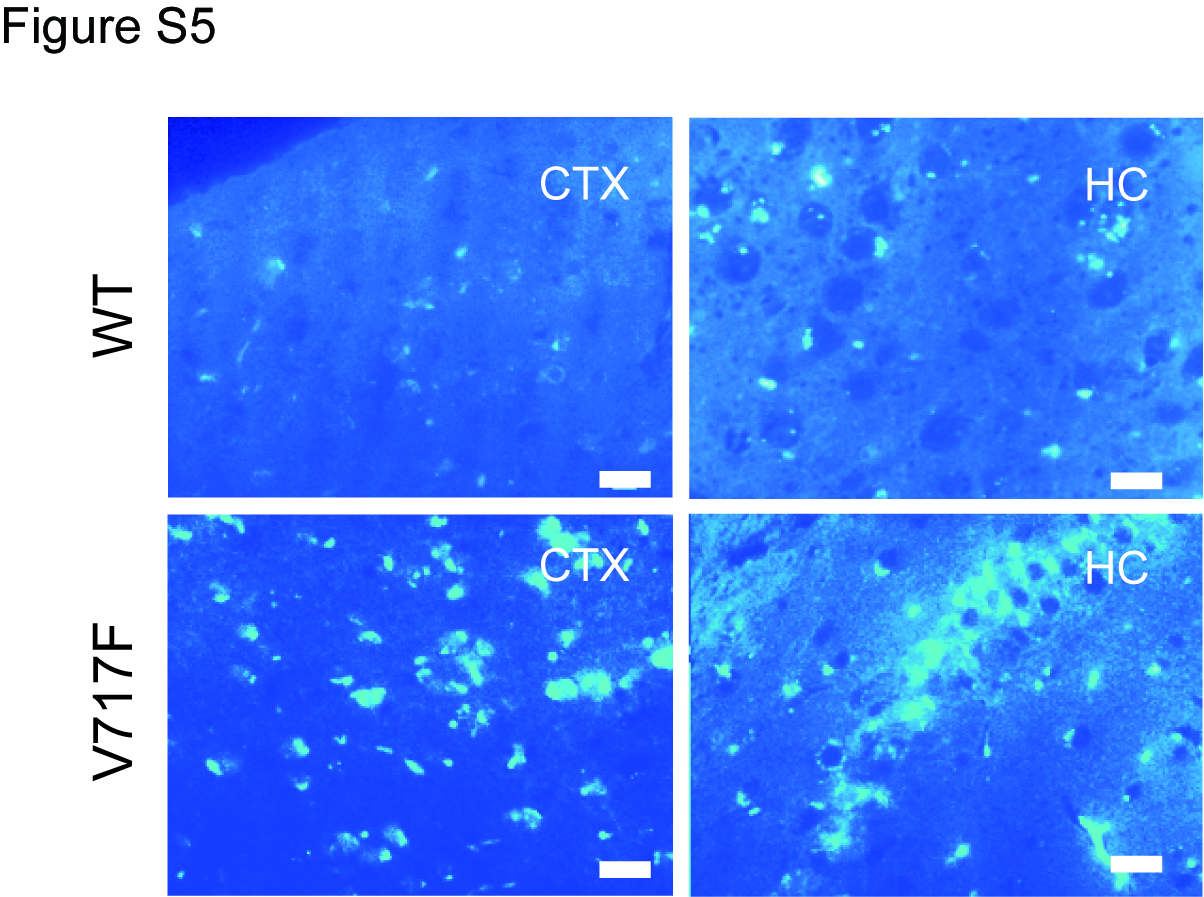

Supplement: Figure S5 — Enlarged images of FSB staining. Sections of the cerebral cortex of the parietal lobe (CTX) and the hippocampus (HC), from 16-month old mice, were stained by FSB. Scale bars, 20 µm. Stronger FSB signals were observed in the sections from water-drinking V717F mice (V717F) than those from age-matched wild-type mice (WT). (TIFF) [file pone.0087185.s005.tiff]
